# In Vitro Comparison of Trueness and Precision of an AI-Driven Real-Time Library Matching Protocol with Irregular Geometry Scan Bodies for Full-Arch Implant Scanning

**DOI:** 10.3390/dj13110533

**Published:** 2025-11-13

**Authors:** Adam Brian Nulty, Cameron Kelly, Oliver Ambridge, Mark Ambridge, Rick Ferguson, Ashtyn Hoffer

**Affiliations:** 1 International Digital Dental Academy, Norwich NR14 8FB, UKollie@ambridgeceramics.com (O.A.); mark@ambridgeceramics.com (M.A.); drferguson@aol.com (R.F.); ashtyn@dentalexcellence.io (A.H.); 2 School of Dentistry, University of Leeds, Leeds LS2 9JT, UK

**Keywords:** intraoral scanners, photogrammetry, edentulous arch, dental implants, Scan Ladder system, accuracy and precision, full arch scanning, All On X

## Abstract

**Background**: Accurate digital transfer of implant positions is critical for the long-term success of full-arch prosthetic rehabilitation. Photogrammetry remains the benchmark for accuracy, but its high cost and complexity limit clinical adoption. Artificial intelligence (AI)-driven intraoral scanning protocols incorporating real-time library matching and irregular, individually coded scan bodies have been proposed as accessible alternatives to improve accuracy and reproducibility. **Methods**: This in vitro study evaluated the trueness and precision of a full-arch implant scanning workflow using an AI-assisted real-time library matching system in combination with irregular multi-geometry titanium scan bodies. A high-accuracy structured-light scanner served as the reference standard. Six implant positions (35, 33, 31, 41, 43, 45) were scanned across 20 datasets (n = 120). Mean surface deviations were calculated against the reference STL using CloudCompare v.2.14. and a two-way ANOVA (α = 0.05) in SPSS tested the effects of implant position and scan iteration. **Results**: The workflow achieved a mean deviation of 13.55 ± 9.70 μm (range 0.77–43.46 μm) across all positions. Anterior sites showed the lowest deviations (e.g., position 31: 3.95 μm; 45: 5.96 μm), while posterior sites exhibited higher deviations (e.g., position 43: 26.15 μm). No mean deviation exceeded 30 μm, and no individual measurement surpassed 45 μm. Implant position significantly affected accuracy (*p* < 0.001), whereas scan iteration did not (*p* > 0.05). **Conclusions:** Within the limitations of this in vitro model, an AI-assisted real-time library matching workflow used in conjunction with irregular multi-geometry scan bodies achieved accuracy levels well within clinically acceptable ranges for full-arch implant impressions. Although comparable to values reported for photogrammetry under laboratory conditions, clinical equivalence should not be assumed. Further in vivo validation is required to confirm performance under routine clinical conditions.

## 1. Introduction

The fabrication of a full-arch implant-supported prosthesis involves multiple steps, each of which may introduce errors that compromise the final accuracy and fit of the restoration. Among these, the precise recording of implant positions is critical and has traditionally depended on impression material, technique, implant depth, angulation, and distribution [1]. Conventional methods, such as open- and closed-tray impressions with polyvinyl siloxane or polyether, remain widely used, often supplemented by splinting to enhance stability [2,3]. Digital impression techniques with intraoral scanners (IOS) have been introduced as an alternative, offering improved efficiency and patient comfort [4,5,6]. Nevertheless, systematic reviews have shown that conventional splinted techniques may still yield superior accuracy in certain situations, particularly in complex edentulous arches [7,8,9,10].

IOS-based workflows have become increasingly common in implant dentistry, providing advantages such as streamlined communication with laboratories and reduced chairside time [11]. While splinted open-tray impressions remain the clinical reference standard, several studies suggest that IOS can achieve comparable accuracy for both partially and fully edentulous implant cases [12,13]. However, the accuracy of IOS in full-arch implant scenarios may be influenced by arch morphology, implant spacing, scanning sequence, ambient conditions, scanner calibration, and scan body design [14,15,16].

Photogrammetry has been established as a reliable digital method for full-arch implant impression accuracy, achieving trueness and precision that often surpass conventional IOS [17]. Despite this, clinical adoption remains limited due to high cost, complexity, and the learning curve associated with photogrammetric workflows [18]. In response, artificial intelligence (AI)-driven scanning protocols incorporating real-time library matching and automated scan body recognition have been developed to improve accessibility and efficiency in digital implant workflows [19].

**Figure 1:**
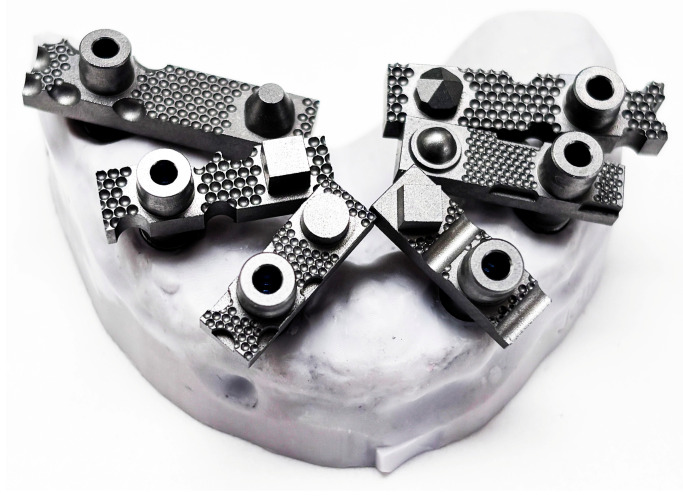
The Scan Ladder Titanium Direct Scan Body Set.

In these systems, the scanner software digitally recognises the scan bodies during acquisition and replaces them with idealised virtual library components in real time, thereby minimising operator-dependent variability and reducing the need for post-processing alignment. When used in combination with multi-geometry, standardised scan bodies (Scan Ladder) designed to optimise recognition and calibration (Figure 1), this workflow may enhance accuracy in full-arch implant scanning. Such scan bodies employ distinct geometries, variable heights, and horizontal alignment to centralise positioning and reduce cumulative stitching error [20].

**Figure 2:**
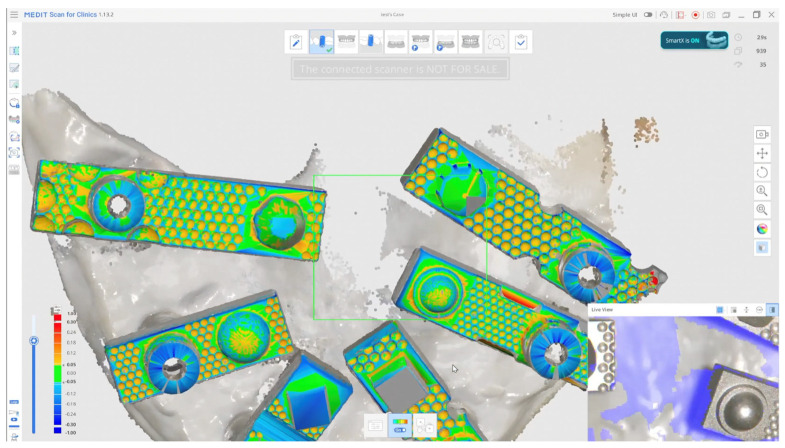
AI-Driven Real-Time Library Matching Protocol with Irregular Geometry Scan Bodies for Full-Arch Implant Scanning; (The green and coloured parts of the image shows the AI data match described in the text).

Although in vitro studies have reported promising outcomes with AI-assisted library matching workflows using standardised multi-geometry scan bodies [21], no published data to date have directly compared such a protocol against a validated laboratory reference scanner in a full-arch implant model. This study addresses this gap by evaluating the trueness and precision of an AI-assisted real-time library matching intraoral scanning workflow in vitro, using a 3Shape E2 structured-light scanner as the reference (Figure 2).

The objective of this investigation was to quantify the accuracy of this workflow in replicating implant positions in terms of trueness and precision, as defined by ISO 5725-1:1994 [22,23,24]. The null hypothesis (H_0_) was that no significant difference would exist between implant positions recorded by the intraoral scanning workflow and those obtained with the reference laboratory scanner. The alternative hypothesis (H_1_) was that the AI-assisted workflow would demonstrate significantly different accuracy compared with the reference.

## 2. Materials and Methods

### 2.1. Model Preparation

This in vitro investigation used a custom-fabricated mandibular full-arch reference model designed to simulate an edentulous clinical scenario. Six implant analogues were embedded to replicate a typical All-on-X configuration at positions 35, 33, 31, 41, 43, and 45. Multi-unit abutments (MUAs) (IPD, San Giuliano Milanese, Italy) were placed on each analogue, and standardised multi-geometry titanium scan bodies (Scan Ladder, Norwich, UK) were connected in their definitive orientation to enable accurate digital identification during scanning.

To ensure reproducible analogue positioning, a verification titanium bar jig was used during model fabrication to control spacing and alignment. A silicone gingival mask was incorporated to reproduce soft-tissue contours and introduce clinically relevant scanning challenges such as subgingival emergence profiles and limited access around angled abutments.

A total of 20 independent intraoral scans were acquired using an AI-assisted intraoral scanning workflow featuring real-time library matching (Medit i900 Scanning Software, Republic of Korea—Smart X Scanning protocol). Each dataset captured all six scan-body positions. The resulting datasets were compared with a high-precision laboratory reference scan generated using a structured-light desktop scanner (3Shape E2; 3Shape, Copenhagen, Denmark).

The study adhered to ISO 12836:2015 standards [25] for in vitro evaluation of scanner trueness and precision. Because no human or animal subjects were involved, ethical approval was not required.

### 2.2. Master STL Creation

The reference dataset was established using the same mandibular model described above. The model, incorporating six implant analogues restored with multi-unit abutments (MUAs) and standardised multi-geometry titanium scan bodies, was digitised with a structured-light desktop scanner (3Shape E2; 3Shape, Copenhagen, Denmark). This device is accredited under ISO 12836:2015 for dental metrology and has a reported trueness specification of ≤7 μm, making it an appropriate reference system for accuracy assessment.

**Figure 3:**
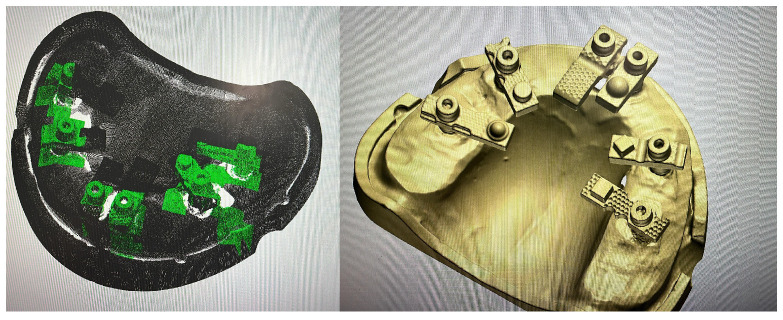
Generation of the Master STL using a structured-light desktop scanner (3Shape E2).

The resulting dataset, exported in STL format, was designated as the Master STL (Figure 3). This file represented the validated reference geometry against which all intraoral scan datasets were subsequently aligned and evaluated.

The purpose of creating the Master STL was to provide a stable, high-fidelity standard for evaluating trueness (closeness of agreement between AI-Driven Real-Time Library Matching Protocol with Irregular Geometry Scan Bodies for Full-Arch Implant scans and the reference) and precision (consistency of repeated AI-Driven Real-Time Library Matching Protocol with Irregular Geometry Scan Bodies for Full-Arch Implant Scanning scans). By anchoring comparisons to this reproducible reference, the study sought to quantify the accuracy of the AI-Driven Real-Time Library Matching Protocol with Irregular Geometry Scan Bodies under controlled in vitro conditions.

### 2.3. Scanning Procedure

The Master STL served as a stable, high-fidelity reference for evaluating trueness—the closeness of agreement between the AI-assisted intraoral scans and the reference—and precision, defined as the consistency among repeated intraoral scans. Anchoring comparisons to this reproducible reference allowed the study to quantify accuracy under controlled in vitro conditions, in accordance with international metrological standards.

The scanning procedure followed the manufacturer’s recommended guidelines for centralised positioning of scan bodies and a standardised scan path across the arch. Scan bodies were positioned along a horizontal plane to maintain consistent reference centroids, thereby minimising potential distortion during data stitching and ensuring balanced spatial distribution.

Each acquisition followed a fixed sequence: scanning began in the right posterior quadrant and progressed sequentially across the occlusal surfaces to the contralateral side, followed by systematic sweeps along the buccal and lingual surfaces. This approach ensured comprehensive capture of soft-tissue contours and full visibility of the scan bodies, facilitating reliable AI-based library recognition within the real-time matching workflow.

**Figure 4:**
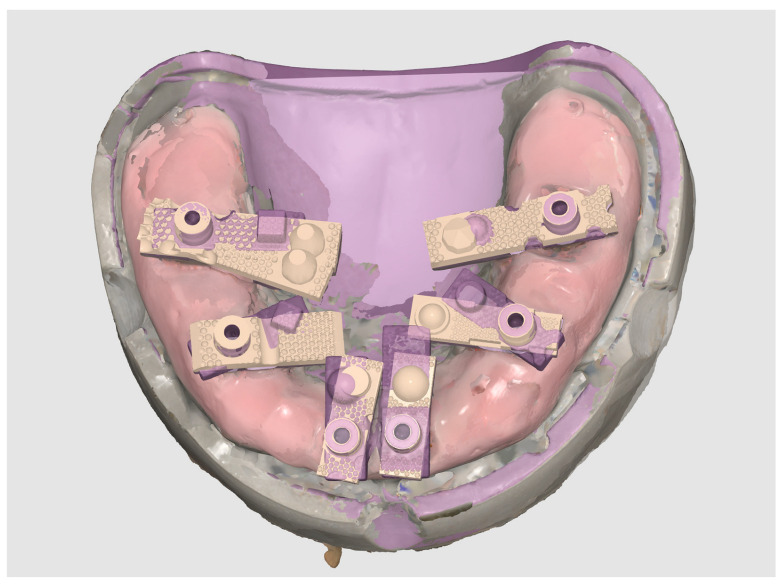
Reference and test datasets aligned within the scanning software, showing consistent overlay and full-arch coverage across all acquisitions.

A total of 20 independent full-arch scans were obtained under identical environmental and hardware conditions by a single experienced operator. During acquisition, the system’s real-time library matching algorithm automatically replaced the physical scan body geometries with corresponding virtual Multi-Unit Abutments (MUAs) within the software environment. Once all scans were completed, the scans were aligned using a best fit match in cloud compare as seen in Figure 4.

**Figure 5:**
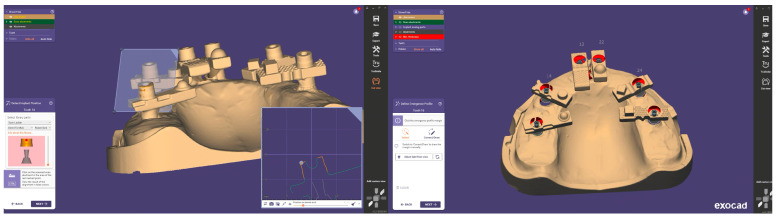
Alignment and segmentation of virtual abutment components within Exocad to generate implant-specific STL datasets.

Each finalised dataset was exported in STL format and processed using Exocad DentalCAD (Exocad GmbH, Darmstadt, Germany). Within Exocad, the six virtual MUAs were digitally isolated and exported as individual files, generating a total of 120 implant-specific STL datasets (20 per site). Figure 5 shows the process of aligning the scan body implant library components in Exocad for the generation of position-specific datasets.

Accuracy evaluation was conducted by importing each test STL and the Master STL (reference scan generated by the structured-light scanner; see Section 2.2) into CloudCompare (open-source software originally developed at Télécom ParisTech/EDF R&D, France, licenced under GPL). Importantly, no iterative closest point (ICP) or best-fit superimposition was applied at this stage. All analyses were performed using the original exported spatial orientations to ensure the positional integrity of each dataset.

For each implant site, the mean surface deviation (µm) was calculated between the test scan and the reference dataset. This yielded one deviation value per implant site per scan—producing 20 values per position and 120 total measurements across the dataset.

**Figure 6:**
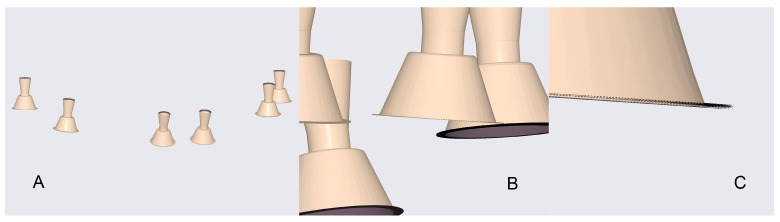
Overlay visualisation of all test datasets: (**A**) full model view; (**B**) close-up of MUA positions; (**C**) detailed view of individual site superimpositions.

Figure 6 provides a visual example of these comparisons, displaying overlays of all datasets: (A) the complete arch view, (B) a magnified view of the MUA positions, and (C) a close-up of individual site superimpositions used for deviation analysis.

#### Scanners in the Study

The scanners used in the present in vitro study are summarised in Table 1.

### 2.4. Design of the Study

#### 2.4.1. Overview

This in vitro study evaluated the accuracy of an AI-assisted intraoral scanning workflow (Medit i900; Medit Corp., Seoul, Republic of Korea) incorporating real-time library matching in conjunction with standardised multi-geometry titanium scan bodies. Accuracy was defined according to ISO 5725-1:1994 standards, encompassing both trueness—the closeness of measured values to a reference—and precision—the repeatability of measurements across repeated scans.

A high-accuracy Master STL reference dataset was generated using a structured-light desktop scanner (3Shape E2; 3Shape, Copenhagen, Denmark), which is accredited under ISO 12836:2015 and specified with a trueness of ≤7 μm. This dataset served as the gold standard for comparative evaluation.

A total of 20 independent intraoral scans were performed, each capturing six implant positions (35, 33, 31, 41, 43, and 45), yielding 120 deviation measurements in total. This dataset size was sufficient to permit statistical assessment of both systematic error (trueness) and measurement variability (precision) between repeated acquisitions under controlled laboratory conditions.

#### 2.4.2. Hypothesis

The study was designed to test the following hypotheses:

**Null Hypothesis (H0):**  *There is no statistically significant difference between the implant positions recorded using the AI-assisted intraoral scanning workflow (Medit i900 operating with real-time library matching and standardised titanium scan bodies) and those obtained from the Master STL generated by the structured-light reference scanner (3Shape E2)*.

**Alternative Hypothesis (H1):**  *There is a statistically significant difference between the implant positions recorded using the AI-assisted intraoral scanning workflow and those obtained from the Master STL reference dataset*.

### 2.5. Data Processing and Analysis

Following completion of the scanning procedures, each full-arch dataset generated by the intraoral scanner was exported in STL format. Within Exocad DentalCAD (Exocad GmbH, Darmstadt, Germany), the automatically aligned virtual Multi-Unit Abutments (MUAs) were digitally segmented from the model. Each implant site was then exported as an independent STL file, yielding six implant-specific datasets per scan and a total of 120 datasets across all 20 acquisitions. This segmentation ensured that each site could be analysed individually while maintaining consistent arch alignment.

Each implant-specific STL was subsequently compared with the corresponding reference implant position from the Master STL (see Section 2.2) using CloudCompare (open-source software originally developed by Daniel Girardeau-Montaut, Télécom ParisTech/EDF R&D, GPL licence). Importantly, no iterative closest point (ICP) or best-fit superimposition was applied. All comparisons were conducted using the raw exported spatial coordinates to preserve the positional integrity of each dataset.

For every implant site, mean surface deviation (expressed in µm) was calculated between the test dataset and the reference model. This procedure yielded 20 deviation values per implant position, providing a quantitative basis for subsequent statistical evaluation of trueness and precision.

#### 2.5.1. Evaluating Trueness

Trueness was defined as the mean deviation between each test scan and the Master STL, representing how closely the intraoral scanning workflow reproduced the true implant geometry. For each implant site, descriptive statistics (mean, standard deviation, minimum, and maximum) were calculated. Pooled results across all sites provided an overall assessment of trueness for the full-arch dataset.

#### 2.5.2. Evaluating Precision

Precision was defined as the degree of agreement among repeated measurements obtained under identical experimental conditions. For each implant site, variability across the 20 replicate scans was analysed. Lower standard deviation values were interpreted as indicative of higher measurement precision, consistent with ISO 5725-1:1994 definitions.

#### 2.5.3. Statistical Analysis Method

Statistical analyses were conducted using SPSS version 22 (IBM Corp., Chicago, IL, USA). A two-way analysis of variance (ANOVA) was applied to evaluate the effects of implant position and scan iteration on deviation values. The assumptions of normality and homogeneity of variance were verified before analysis. When significant main effects were identified, Tukey’s HSD post hoc test was used to adjust for multiple comparisons. The level of significance was set at α = 0.05.

To enhance analytical robustness, all deviation values were expressed as absolute values, in accordance with ISO 5725-1:1994 recommendations, ensuring that negative deviations did not offset positive ones. This approach provided a reproducible framework for quantifying trueness and precision, and enabled direct comparison with data from previous studies on intraoral scanning and photogrammetry accuracy.

## 3. Results

A total of 120 measurements were analysed, representing six implant positions as shown in Table 2 (35, 33, 31, 41, 43, and 45) across 20 full-arch scans acquired with the intraoral scanner operating under the AI-driven real-time library matching protocol. All datasets were compared against the corresponding reference positions obtained from the 3Shape E2 desktop structured-light scanner. The Master STL, produced using a verification jig, was regarded as the reference standard for all trueness and precision assessments.

### 3.1. Trueness

Across all implant sites, the workflow demonstrated a mean deviation of 13.55 ± 9.70 μm (range: 0.77–43.46 μm). These findings indicate that the system achieved deviations substantially below the commonly referenced clinical tolerance of ±150 μm for full-arch implant impressions.

At the individual implant level, the lowest mean deviations were observed at position 31 (4.95 ± 3.17 μm; range 0.85–12.77 μm) and position 45 (5.96 ± 3.64 μm; range 0.77–14.07 μm). The highest mean deviation occurred at position 43 (26.15 ± 8.68 μm; range 12.10–43.46 μm). Although posterior sites exhibited slightly greater variability than anterior sites, no mean deviation exceeded 30 μm at any position.

**Figure 7:**
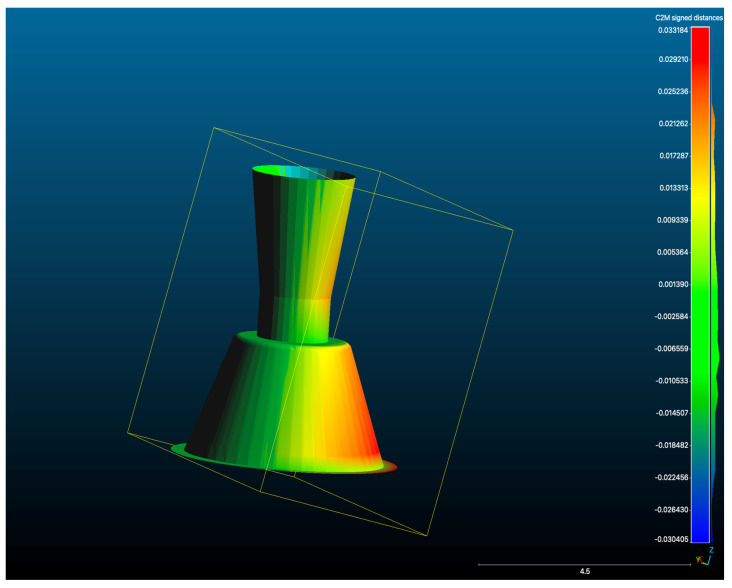
Overlay of a representative test scan STL with the Master STL, illustrating deviation mapping between the two datasets (μm).

**Figure 8:**
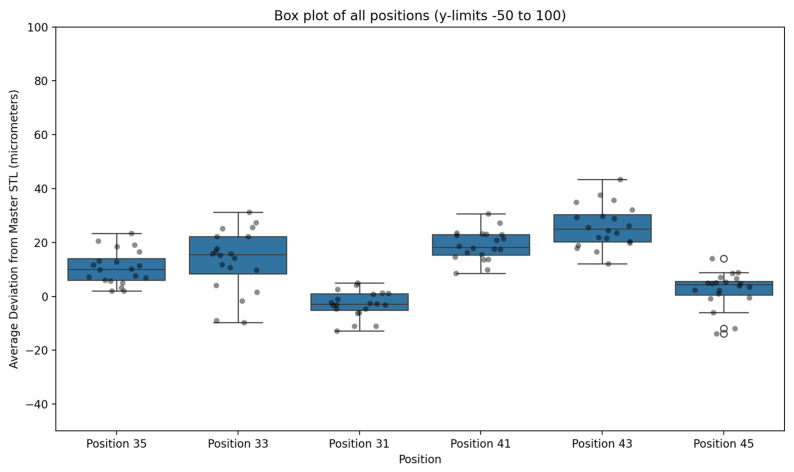
Box plot illustrating deviation values (μm) for each implant position across 20 full-arch scans acquired using the AI-driven real-time library matching protocol, compared with the Master STL reference. Outliers are expressed with an circles.

Figure 7 illustrates an overlay of a representative test STL and the Master STL, visualising the localised deviations. Table 3 summarises trueness values for each implant site, while Figure 8 presents a box plot of deviation distributions.

### 3.2. Precision

Repeated scans demonstrated a high degree of reproducibility. The pooled dataset produced a standard deviation of 9.70 μm, indicating low variability across the 20 replicate scans per implant site. Anterior positions exhibited particularly consistent measurements (e.g., position 31: SD 3.17 μm; position 45: SD 3.64 μm), whereas posterior sites showed slightly greater variability (e.g., position 43: SD 8.68 μm). Despite these differences, all positions remained well within clinically acceptable limits for full-arch implant impression accuracy.

### 3.3. Statistical Analysis

The two-way ANOVA demonstrated that implant position significantly influenced deviation values (*p* < 0.001). Posterior implants exhibited higher mean deviations than anterior sites, with the greatest discrepancies observed at position 43.

In contrast, scan iteration showed no significant effect (*p* > 0.05), indicating consistent performance across repeated scans. No significant interaction was identified between implant position and scan iteration (*p* > 0.05), suggesting that the observed deviations were primarily anatomical in nature—likely related to arch span and posterior angulation—rather than operator- or sequence-dependent.

Table 4 summarises the compared means and post hoc Tukey subsets (α = 0.05), confirming the statistical significance of positional differences.

## 4. Discussion

### 4.1. Evaluation of Trueness and Precision in Digital Intraoral Scanners

The aim of this in vitro investigation was to evaluate the trueness and precision of full-arch implant scans acquired using an AI-assisted real-time library matching protocol in combination with irregular multi-geometry titanium scan bodies. A structured-light desktop scanner served as the reference standard.

Across all implant positions, the workflow demonstrated a mean absolute deviation of 13.55 ± 9.70 μm (range: 0.77–43.46 μm), indicating that the deviations observed were substantially below the generally accepted clinical tolerance of approximately 150 μm for passive fit in full-arch implant prosthodontics.

Statistical analysis showed that implant position significantly influenced deviation values (*p* < 0.001), while scan iteration had no measurable effect (*p* > 0.05), confirming strong repeatability under identical conditions. Posterior sites presented slightly higher deviation magnitudes than anterior positions, consistent with previous reports that increased scanning span, inter-implant distance, and posterior angulation can amplify accumulated error during intraoral digitisation [14,15,16].

Although the workflow achieved deviation levels comparable to those reported for laboratory photogrammetry in analogous in vitro studies [17,18,19], intraoral variables—such as saliva, patient movement, and restricted optical access—were not simulated. Accordingly, direct clinical equivalence to photogrammetry cannot be inferred. Instead, these findings suggest that AI-driven real-time library matching, when integrated with irregular, multi-geometry scan body design, can achieve reproducible accuracy within clinically acceptable limits for full-arch digital implant workflows [20,21,22,23,26,27,28,29].

### 4.2. Contribution to Existing Scientific Literature

The findings of this study contribute to the growing body of evidence on the accuracy of artificial intelligence (AI)-assisted digital workflows for full-arch implant impression transfer. Previous investigations have consistently demonstrated that photogrammetry provides highly reliable spatial reproduction of implant positions, typically reporting mean deviations below 20 μm under controlled laboratory conditions [20,30,31]. However, its widespread clinical use remains constrained by factors such as system cost, limited equipment availability, and the need for operator training [32,33,34].

The AI-assisted real-time library matching protocol evaluated in this study represents a software-based alternative that automates the recognition and alignment of virtual scan body geometries during intraoral scanning. When combined with irregular multi-geometry titanium scan bodies, the system reduces the reliance on manual library alignment or external calibration devices.

The present in vitro results indicate that this AI-driven workflow can achieve accuracy values of a similar magnitude to those reported for photogrammetry under laboratory conditions. Nevertheless, these findings should be interpreted cautiously, as direct clinical equivalence cannot be inferred without complementary in vivo validation accounting for biological variability, operator movement, and intraoral environmental factors.

### 4.3. Enhancement in Accuracy Through Scan-Body Geometry

Scan-body design is a well-recognised determinant of intraoral-scanner accuracy [35,36,37,38,39]. In this study, a system of irregular, multi-geometry titanium scan bodies was used to optimise scan capture within an AI-assisted real-time library-matching protocol. The scan bodies feature horizontally aligned centroids and distinctive, non-repetitive surface geometries intended to improve digital recognition and reduce angular distortion between implants.

This geometric configuration facilitates alignment by the AI algorithm, enhances detection of individual markers, and allows adjustment of scan-body positioning relative to local anatomy. Collectively, these design principles aim to reduce cumulative stitching error and improve data capture in areas of limited access, such as tilted or posterior implants with extended edentulous spans. The results of this study suggest that such features contributed to the low deviation values observed across the arch.

### 4.4. Comparison with Peer-Reviewed Literature

Previous investigations have highlighted the influence of scan-body morphology and implant angulation on digital-impression accuracy. Arcuri et al. reported positional discrepancies exceeding 100 μm in certain full-arch configurations, particularly at posterior or angled sites [19]. In contrast, the present study recorded mean deviations below 30 μm across all implant positions. This improvement likely reflects the combined effects of AI-assisted real-time library matching and the irregular surface geometry of the scan bodies used.

Similarly, Gómez-Polo et al. demonstrated that optimised scan-body design enhances trueness in digital impressions [36]. The present findings are consistent with those observations, reinforcing the principle that precise integration of scan-body geometry with advanced scanning algorithms can significantly improve the fidelity of full-arch digital implant workflows.

### 4.5. Implications for Clinical Practice

A key observation from this study was that scan iteration had no significant effect on accuracy, indicating high reproducibility of the AI-assisted real-time library matching workflow under standardised conditions. Because this system operates within an established intraoral-scanner software environment, implementation requires minimal additional calibration or operator training, which could lower the barrier to clinical integration.

Nevertheless, these findings were derived from an in vitro model, and clinical application should be approached with caution. Biological and environmental variables—such as saliva, patient movement, and limited intraoral access—were not simulated in the controlled laboratory setting and may influence performance in vivo.

### 4.6. Challenges and Limitations of the Present Study

Several limitations must be acknowledged. First, the study’s in vitro design restricts direct extrapolation to clinical contexts. Although a silicone gingival mask was included to replicate soft-tissue interference, dynamic biological effects were not assessed. Second, the reference scan (E2, 3Shape, Copenhagen, Denmark) was acquired once; repeated reference scans would have enabled quantification of the reference device’s inherent variability. Finally, all deviation values were reported as absolute magnitudes. While this approach prevents cancellation of positive and negative errors, it may slightly overestimate local mean deviations. These factors should be considered when interpreting the outcomes.

### 4.7. Future Directions

Further research should include in vivo validation to assess how AI-assisted real-time library matching performs under routine clinical conditions when combined with irregular, multi-geometry scan bodies. Larger sample sizes and comparative trials against other AI-driven intraoral-scanning systems and photogrammetry platforms will help contextualise accuracy among different technologies. Additional investigations into the effects of implant angulation, inter-implant spacing, and repeated reuse of scan bodies would provide valuable insights into long-term consistency and clinical reliability.

## 5. Conclusions

This in vitro investigation demonstrated that an AI-assisted real-time library matching protocol, when used in combination with irregular, multi-geometry titanium scan bodies, achieved a mean absolute deviation of 13.55 ± 9.70 μm, with no individual implant site exceeding 30 μm. These results indicate a high level of trueness and precision relative to a validated laboratory-grade structured-light scanner, and remain well within ranges generally regarded as clinically acceptable for full-arch implant impression accuracy.

The findings suggest that integrating AI-based library matching with an optimised scan-body geometry can provide a streamlined and reproducible digital workflow for full-arch implant scanning. The geometric attributes of the scan bodies—such as variable height, irregular morphology, and horizontally standardised positioning—likely contributed to the observed performance under controlled laboratory conditions.

However, these results should be interpreted cautiously, as the study was performed entirely in vitro. Biological and clinical variables, including saliva, soft-tissue movement, and restricted intraoral access, were not represented. Consequently, direct clinical equivalence to photogrammetry or other high-precision systems cannot be inferred. Further in vivo validation and comparative trials against alternative AI-assisted and photogrammetric workflows are warranted to confirm clinical applicability and long-term reliability.

Within these boundaries, the present study provides preliminary evidence supporting the accuracy and repeatability of AI-assisted real-time library matching using multi-geometry scan bodies and highlights the importance of continued research to establish its clinical performance in routine implant rehabilitation.

## Figures and Tables

**Table 1:** The Digital Scanners Used In This Study.

Name	Manufacturer	Technology	STL Export	PLY/OBJ Colour Export	Photogrammetry
E2	3Shape, Copenhagen, Denmark	Structured white light desktop scanner.	YES	NO	NO
i900	Medit, Seongbuk-gu, Seoul, Republic of Korea	Structured light-Active Speed 3D Video™	YES	YES	NO

**Table 2:** Summary of Deviation Measurements (μm) across All Implant Positions.

Block Number	Position 35	Position 33	Position 31	Position 41	Position 43	Position 45
1	3.18	25.12	3.19	23.06	26.17	0.98
2	18.43	15.23	4.7	8.58	19.86	4.79
3	10.10	31.28	6.33	17.42	43.46	7.05
4	12.87	4.05	2.61	13.70	18.93	3.54
5	9.92	15.77	2.75	30.69	23.54	5.96
6	7.25	9.75	0.85	9.84	32.06	4.93
7	4.91	22.25	4.25	13.59	34.90	5.26
8	7.71	22.24	1.27	21.42	12.10	0.77
9	11.36	1.70	1.17	23.19	25.56	14.07
10	16.52	10.81	6.07	18.65	24.36	4.78
11	20.62	16.73	3.36	16.20	21.85	8.47
12	6.08	8.94	4.71	22.88	21.64	3.87
13	19.00	14.21	10.99	23.50	16.48	5.15
14	6.92	15.82	1.14	14.63	37.62	6.59
15	2.04	17.68	12.77	15.54	17.84	13.88
16	5.67	27.43	2.26	20.90	35.72	2.10
17	2.04	25.55	5.00	17.62	29.29	11.88
18	11.68	11.77	3.16	22.60	29.70	0.56
19	23.44	1.53	11.08	27.17	28.85	8.81
20	13.16	9.78	2.62	17.86	20.41	2.32

**Table 3:** Mean Deviation (μm) and Standard Deviation per Implant Position Compared with Master STL (3Shape E2).

Name	Mean (μm)	Std. Deviation (μm)	*p* Value
Position 35	10.65	6.32	<000.1
Position 33	15.38	8.41	<000.1
Position 31	4.54	3.54	<000.1
Position 41	18.95	5.57	<000.1
Position 43	26.02	7.94	<000.1
Position 45	5.79	3.99	<000.1

**Table 4:** Inter-Position Correlation and Comparative Analysis of Deviations (N = 20).

Position	N Complete	Mean Corr to Others	Max Corr to Other	Max Corr Partner	Mean Abs Diff to Others	Best Linear R2	Best Linea Partner
Position 45	20	−0.08	0.26	Position 35	10.21	0.26	Position 35
Position 43	20	−0.09	0.36	Position 33	10.90	0.26	Position 33
Position 33	20	0.12	0.40	Position 43	11.63	0.16	Position 43
Position 35	20	0.00	0.16	Position 45	11.10	0.02	Position 45
Position 31	20	−0.03	0.36	Position 35	15.71	0.13	Position 35
Position 41	20	0.05	0.40	Position 31	11.03	0.16	Position 31

## Data Availability

The original contributions presented in the study are included in the article material, further inquiries can be directed to the corresponding author.

## References

[1] Zafiropoulos G.-G., Galil A.A., Deli G. (2014). An Interocclusal Recording Method for the Fabrication of Full-Arch Implant-Retained Restorations. J. Oral Implantol..

[2] Jain M. (2015). Impression Techniques for the Resorbed Mandibular Arch: A Guide to Increased Stability. J. Sci. Soc..

[3] Leão M.P., Pinto C.P., Sponchiado A.P., Ornaghi B.P. (2014). Dimensional Stability of a Novel Polyvinyl Siloxane Impression Technique. Braz. J. Oral Sci..

[4] Rutkūnas V., Gečiauskaitė A., Jegelevičius D., Vaitiekūnas M. (2017). Accuracy of Digital Implant Impressions with Intraoral Scanners: A Systematic Review. Eur. J. Oral Implantol..

[5] Di Fiore A., Meneghello R., Graiff L., Savio G., Vigolo P., Monaco C., Stellini E. (2019). Full-Arch Digital Scanning Systems Performances for Implant-Supported Fixed Dental Prostheses: A Comparative Study of Eight Intraoral Scanners. J. Prosthodont. Res..

[6] Ender A., Mehl A. (2013). Influence of Scanning Strategies on the Accuracy of Digital Intraoral Scanning Systems. Int. J. Comput. Dent..

[7] Drancourt N., Auroy P., Veyrune J.-L., Osta N.E., Nicolas E. (2023). Accuracy of Conventional and Digital Impressions for Full-Arch Implant-Supported Prostheses: An In Vitro Study. J. Pers. Med..

[8] Fayad N.E.H., Abofadle A., Bahig D. (2024). Accuracy of Different Digital Data Acquisition Workflows for Full-Arch Maxillary Implant Prostheses: An In-Vitro Study. Egypt. Dent. J..

[9] Rutkūnas V., Gedrimiene A., Adaškevičius R., Al-Haj Husain N., Özcan M. (2020). Comparison of the Clinical Accuracy of Digital and Conventional Dental Implant Impressions. Eur. J. Prosthodont. Restor. Dent..

[10] Papaspyridakos P., Vazouras K., Chen Y.-W., Kotina E., Natto Z.S., Kang K., Chochlidakis K. (2020). Digital vs. Conventional Implant Impressions: A Systematic Review and Meta-Analysis. J. Prosthodont..

[11] Rahman R.N., Lee A., Lavasani S., Boehm T. (2022). An Overview of Digital Workflows for Precision Implant Dentistry. J. Calif. Dent. Assoc..

[12] Miyoshi K., Tanaka S., Yokoyama S., Sanda M., Baba K. (2020). Effects of Different Types of Intraoral Scanners and Scanning Ranges on the Precision of Digital Implant Impressions in the Edentulous Maxilla: An In-Vitro Study. Clin. Oral Implants Res..

[13] Mangano F., Veronesi G., Hauschild U., Mijiritsky E., Mangano C. (2016). Trueness and Precision of Four Intraoral Scanners in Oral Implantology: A Comparative In-Vitro Study. PLoS ONE.

[14] Tallarico M., Lumbau A.I., Scrascia R., Demelas G., Sanseverino F., Amarena R., Meloni S.M. (2020). Feasibility of Using a Prosthetic-Based Impression Template to Improve the Trueness and Precision of a Complete-Arch Digital Impression on Four and Six Implants: An In-Vitro Study. Materials.

[15] Sultanoğlu E.G., Keleş B. (2024). Comparison of the Accuracy and Precision of Digital Scans for Implant-Supported Maxillary Hybrid Prostheses: An In-Vitro Study. Niger. J. Clin. Pract..

[16] Giménez B., Özcan M., Martínez-Rus F., Pradíes G. (2015). Accuracy of a Digital Impression System Based on Active Wavefront Sampling Technology for Implants Considering Operator Experience, Implant Angulation, and Depth. Clin. Implant Dent. Relat. Res..

[17] Cheng J., Zhang H., Liu H., Li J., Wang H.-L., Tao X. (2024). Accuracy of Edentulous Full-Arch Implant Impression: An In-Vitro Comparison between Conventional Impression, Intraoral Scan with and without Splinting, and Photogrammetry. Clin. Oral Implants Res..

[18] Çakmak G., Yilmaz H., Treviño A., Kökat A.M., Yilmaz B. (2020). The Effect of Scanner Type and Scan Body Position on the Accuracy of Complete-Arch Digital Implant Scans. Clin. Implant Dent. Relat. Res..

[19] Arcuri L., Pozzi A., Lio F., Rompen E., Zechner W., Nardi A. (2020). Influence of Implant Scan-Body Material, Position and Operator on the Accuracy of Digital Impressions for Complete-Arch Restorations: A Randomised In-Vitro Trial. J. Prosthodont. Res..

[20] Örtorp A., Jemt T., Bäck T. (2005). Photogrammetry and Conventional Impressions for Recording Implant Positions: A Comparative Laboratory Study. Clin. Implant Dent. Relat. Res..

[21] Rivara F., Lumetti S., Calciolari E., Toffoli A., Forlani G., Manfredi E. (2016). Photogrammetric Method to Measure the Discrepancy between Clinical and Software-Designed Positions of Implants. J. Prosthet. Dent..

[22] Richert R., Goujat A., Venet L., Viguie G., Viennot S., Robinson P., Farges J.-C., Fages M., Ducret M. (2017). Intraoral Scanner Technologies: A Review to Make a Successful Impression. J. Healthc. Eng..

[23] Vafaee F., Firouz F., Mohajeri M., Hashemi R., Ghorbani Gholiabad S. (2021). In-Vitro Comparison of the Accuracy (Precision and Trueness) of Seven Dental Scanners. J. Dent..

[24] (1994). Accuracy (Trueness and Precision) of Measurement Methods and Results—Part 1: General Principles and Definitions.

[25] (2015). Dentistry—Digitizing Devices for CAD/CAM Systems for Indirect Dental Restorations—Test Methods for Assessing Accuracy.

[26] Nulty A.B. (2024). An In-Vivo Comparison of Trueness and Precision of Two Novel Methods for Improving Edentulous Full-Arch Implant Scanning Accuracy: A Pilot Study. Dent. J..

[27] Menditto A., Patriarca M., Magnusson B. (2007). Understanding the Meaning of Accuracy, Trueness and Precision. Accredit. Qual. Assur..

[28] Bonnick S.L., Lewis L.A. (2013). The Importance of Precision. Positioning Techniques in Clinical Dentistry.

[29] Prenesti E., Gosmaro F. (2015). Trueness, Precision and Accuracy: A Critical Overview of the Concepts as Well as Proposals for Revision. Accredit. Qual. Assur..

[30] Bratos M., Bergin J.M., Rubenstein J.E., Sorensen J.A. (2018). Effect of Simulated Intraoral Variables on the Accuracy of a Photogrammetric Imaging Technique for Complete-Arch Implant Prostheses. J. Prosthet. Dent..

[31] Bergin J.M., Rubenstein J.E., Mancl L., Brudvik J.S., Raigrodski A.J. (2013). An In-Vitro Comparison of Photogrammetric and Conventional Complete-Arch Implant Impression Techniques. J. Prosthet. Dent..

[32] Azevedo L., Molinero-Mourelle P., Antonaya-Martín J.L., del Río-Highsmith J., Correia A., Gómez-Polo M. (2019). Photogrammetry Technique for the 3D Digital Impression of Multiple Dental Implants. Int. J. Environ. Res. Public Health.

[33] Stuani V.T., Ferreira R., Manfredi G.G.P., Cardoso M.V., Sant’Ana A.C.P. (2019). Photogrammetry as an Alternative for Acquiring Digital Dental Models: A Proof of Concept. Med. Hypotheses.

[34] De Angelis F., Pranno N., Franchina A., Di Carlo V., Brauner E., Ferri A., Pellegrino G., Grecchi E., Goker F., Stefanelli L.V. (2022). Artificial Intelligence: A New Diagnostic Software in Dentistry—A Preliminary Performance Diagnostic Study. Int. J. Environ. Res. Public Health.

[35] Huang R., Liu Y., Huang B., Zhang C., Chen Z., Li Z. (2020). Improved Scanning Accuracy with Newly Designed Scan Bodies: An In-Vitro Study Comparing Digital versus Conventional Impression Techniques for Complete-Arch Implant Rehabilitation. Clin. Oral Implants Res..

[36] Gómez-Polo M., Sallorenzo A., Ortega R., Gomez-Polo C., Barmak A.B., Att W., Revilla-León M. (2022). Influence of Implant Angulation and Clinical Implant Scan Body Height on the Accuracy of Complete-Arch Intraoral Digital Scans. J. Prosthet. Dent..

[37] Ntovas P., Spanopoulou M., Martin W., Sykaras N. (2022). Superimposition of Intraoral Scans of an Edentulous Arch with Implants and Implant-Supported Provisional Restoration Implementing a Novel Implant Prosthetic Scan Body. J. Prosthodont. Res..

[38] Thanasrisuebwong P., Kulchotirat T., Anunmana C. (2021). Effects of Inter-Implant Distance on the Accuracy of Intraoral Scanners: An In-Vitro Study. J. Adv. Prosthodont..

[39] Jemt T., Bäck T., Petersson A. (1999). Photogrammetry—An Alternative to Conventional Impressions in Implant Dentistry? A Clinical Pilot Study. Int. J. Prosthodont..

